# Respiratory Immunization With a Whole Cell Inactivated Vaccine Induces Functional Mucosal Immunoglobulins Against Tuberculosis in Mice and Non-human Primates

**DOI:** 10.3389/fmicb.2020.01339

**Published:** 2020-06-18

**Authors:** Nacho Aguilo, Santiago Uranga, Elena Mata, Raquel Tarancon, Ana Belén Gómez, Dessislava Marinova, Isabel Otal, Marta Monzón, Juan Badiola, Dolores Montenegro, Eugenia Puentes, Esteban Rodríguez, Richard A. W. Vervenne, Claudia C. Sombroek, Frank A. W. Verreck, Carlos Martín

**Affiliations:** ^1^Grupo de Genética de Micobacterias, Universidad de Zaragoza, Zaragoza, Spain; ^2^CIBER Enfermedades Respiratorias, Instituto de Salud Carlos III, Madrid, Spain; ^3^Research Centre for Encephalopathies and Transmissible Emerging Diseases, Universidad de Zaragoza, Zaragoza, Spain; ^4^Biofabri, Porrino, Spain; ^5^Biomedical Primate Research Centre (BPRC), Rijswijk, Netherlands; ^6^Servicio de Microbiología, Hospital Universitario Miguel Servet, ISS Aragón, Zaragoza, Spain

**Keywords:** whole-cell vaccine, pulmonary vaccination, animal models, mucosal immunoglobulins, opsonization, tuberculosis

## Abstract

Vaccination through the natural route of infection represents an attractive immunization strategy in vaccinology. In the case of tuberculosis, vaccine delivery by the respiratory route has regained interest in recent years, showing efficacy in different animal models. In this context, respiratory vaccination triggers lung immunological mechanisms which are omitted when vaccines are administered by parenteral route. However, contribution of mucosal antibodies to vaccine- induced protection has been poorly studied. In the present study, we evaluated in mice and non-human primates (NHP) a novel whole cell inactivated vaccine (MTBVAC HK), by mucosal administration. MTBVAC HK given by intranasal route to BCG-primed mice substantially improved the protective efficacy conferred by subcutaneous BCG only. Interestingly, this improved protection was absent in mice lacking polymeric Ig receptor (pIgR), suggesting a crucial role of mucosal secretory immunoglobulins in protective immunity. Our study in NHP confirmed the ability of MTBVAC HK to trigger mucosal immunoglobulins. Importantly, *in vitro* assays demonstrated the functionality of these immunoglobulins to induce *M. tuberculosis* opsonization in the presence of human macrophages. Altogether, our results suggest that mucosal immunoglobulins can be induced by vaccination to improve protection against tuberculosis and therefore, they represent a promising target for next generation tuberculosis vaccines.

## Introduction

Tuberculosis (TB) disease causes one and a half million deaths per year, and is one of the leading infectious diseases affecting mainly developing and underdeveloped countries. The rising spread of multidrug resistant strains with increasing human migration makes TB an alarming global health problem, according to World Health Organization (WHO). Therefore, there is an urgent need for new effective TB vaccines.

Vaccination through the natural route of infection represents an attractive strategy for priming the natural host immunity. In the case of TB, respiratory mucosal tissue is the primary site for establishment of infection. It has been well described in different preclinical models that vaccination with BCG by the respiratory route confers a substantially improved protection in comparison to subcutaneous or intradermal immunization (Lagranderie et al., 1993; Aguilo et al., 2016; Dijkman et al., 2019). Indeed, in the last few years, it has raised an interest in exploring new vaccination approaches delivered through respiratory routes of administration. These strategies include attenuated *M. tuberculosis* (Kaushal et al., 2015) in addition to BCG, as well as subunit vaccines formulated with adjuvants or non-replicative virus (Stylianou et al., 2015; Woodworth et al., 2019). In 2014, the first clinical trial of an aerosol tuberculosis vaccine was reported (Satti et al., 2014).

It is assumed that inactivation of whole-cell tuberculosis vaccines reduces their immunogenic and protective potential. Nevertheless, and likely based on safety concerns described for live BCG under specific conditions (e.g., immunodeficiencies), researchers have explored the use of inactivated vaccine approaches for tuberculosis. To overcome the loss of immunogenicity, different strategies have been conducted, such as the use of inactivated whole-cell vaccines as booster for BCG (Von Reyn et al., 2017).

The present work describes vaccination with a heat-killed (HK) version of the live attenuated *M. tuberculosis* vaccine MTBVAC (Arbues et al., 2013) both in mice and non-human primates (NHP). MTBVAC is the first and only live attenuated tuberculosis vaccine based on *M. tuberculosis* that has reached clinical stages of development, and it has shown an excellent safety profile both in adults and newborns, as well as stronger immunogenicity compared to BCG (Spertini et al., 2015; Tameris et al., 2019). Results in the present study demonstrate improved efficacy of MTBVAC HK when given by intranasal route to mice previously vaccinated with subcutaneous BCG. In addition, we interrogated lung humoral immune responses elicited by MTBVAC HK in mice and NHP, finding an induction of tuberculosis-specific mucosal immunoglobulins with functional activity against *M. tuberculosis*.

## Results

### Intranasal MTBVAC HK Enhances Protection Conferred by Subcutaneous BCG

**FIGURE 1:**
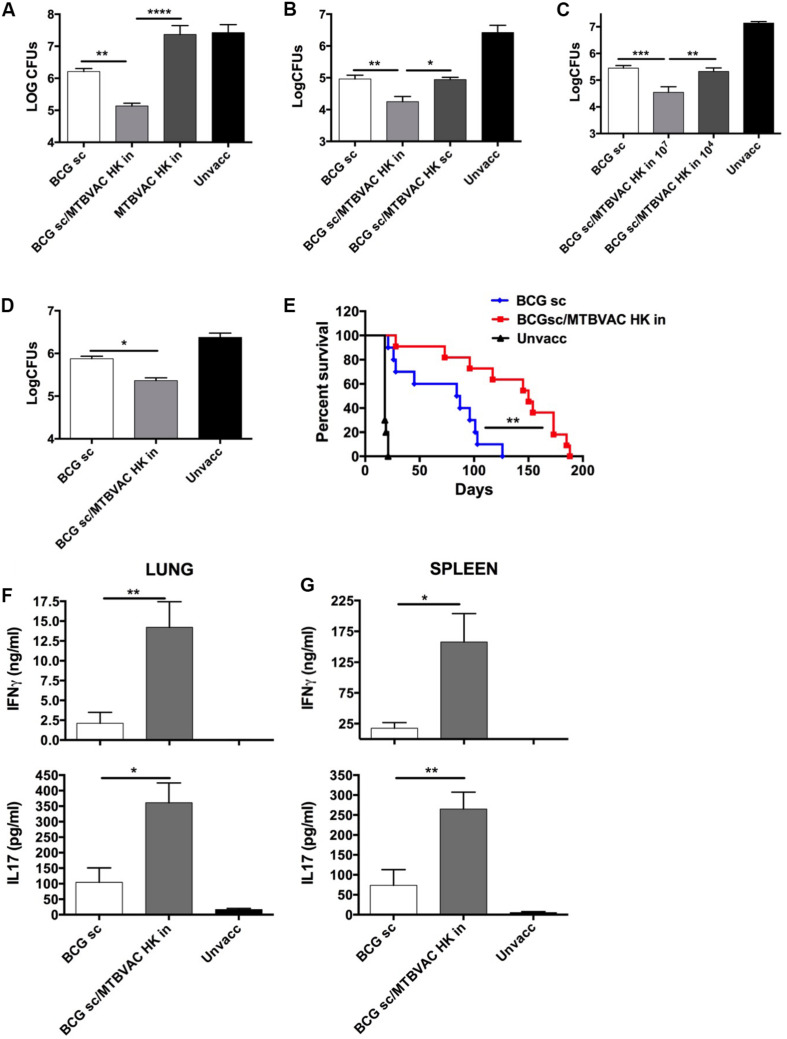
Improved protection induced by intranasal MTBVAC HK as boost of subcutaneous BCG. **(A–C)** Groups of C57BL/6 adult mice where vaccinated with BCG and MTBVAC HK 4 weeks apart (107 MTBVAC HK dose when not specified). After one month, mice were intranasally challenged with H37Rv and lung bacterial load analyzed one month later. **(D,E)** Newborn mice were vaccinated with BCG and 8 weeks later boosted intranasally with 107 MTBVAC HK. Mice were challenged with a low-dose **(D)** or a high-dose **(E)** H37Rv inoculation and lung CFUs or survival analyzed, respectively. **(F,G)** Antigen-specific IFNg and IL17A production one month after MTBVAC HK vaccination, following PPD stimulation of cells from lungs **(F)** and spleen **(G)**. **(A–D, F,G)** Data are shown as mean ± SEM and are representative of at least two independent experiments. (*n* = 6 mice/group). *p < 0.05; **p < 0.001; ***p < 0.001; ****p < 0.0001 by one-way ANOVA and Bonferroni post-test. **(E)** Data from one experiment (*n* = 10 mice/group) are represented in a Kaplan-Meier survival curve and statistical significance calculated by a LogRank test. **p < 0.01.

We and others have previously demonstrated an advantageous vaccine-induced protection of whole-cell live vaccines when given by respiratory route, compared to subcutaneous or intradermal administration in immunologically naïve subjects (Aguilo et al., 2016). Since respiratory airways could be a sensitive organ for exacerbated inflammatory response caused by live bacteria in the pre-exposed, we chose to evaluate the efficacy of an inactivated whole cell vaccine as a booster strategy after primary BCG. To this end, we inactivated MTBVAC building upon the promising immunogenic profile shown by the live version of this vaccine both in animal models and in humans (Marinova et al., 2017). MTBVAC was inactivated by heating the vaccine at 100°C for 30 min. Bacterial inactivation was confirmed by plating on 7H10 solid agar medium (data not shown). Bacteria visualization using electron microscopy confirmed that MTBVAC maintained their bacillary shape upon heat treatment (Supplementary Figure S1). Then, we characterized MTBVAC HK-conferred protection in mice under different experimental conditions. First, 10^7^ MTBVAC HK bacteria were inoculated intranasally in naïve or in BCG-primed mice. One month later, mice were challenged intranasally with a low dose (150 CFU) of the H37Rv Mtb strain. After one month, mice were sacrificed and lung bacterial load evaluated by plating in solid medium. Our data showed that MTBVAC HK alone did not confer protection compared to unvaccinated control. However, when combined with subcutaneous BCG priming, protection was about one-log higher than that provided by BCG only (Figure 1A), indicating the need of a BCG prime to trigger a MTBVAC HK-induced protective response.

We also evaluated protection induced by MTBVAC killed with formalin, since this method of inactivation had been shown in a previous study with other inactivated vaccines to better preserve immunogenicity compared to heating (Cryz et al., 1982). However, in this case, we did not find any difference in efficacy between both MTBVAC inactivation methods (Supplementary Figure S2). Since heat-inactivation is easier to implement, we continued vaccine characterization using this method of inactivation. MTBVAC HK induced better protection only when given by intranasal route, but did not improve BCG when administered subcutaneously (Figure 1B). Ultimately, our data show that the MTBVAC HK booster effect was dose-dependent, as we only observed improved protection with a high dose of MTBVAC HK (10^7^), whereas no effect was obtained using 10^4^ bacteria (Figure 1C). We and others have previously reported the lack of protection induced by BCG subcutaneous in the mouse strain DBA/2 (Aguilo et al., 2016). Interestingly, MTBVAC HK also induced protection in BCG-vaccinated DBA/2 mice, suggesting that this vaccination approach could confer protective efficacy in cases in which BCG is ineffective (Supplementary Figure S3). Comparison of different independent lots of MTBVAC HK provided a similar protective profile, superior to BCG only, evidencing the reproducibility of our results (Supplementary Figure S4).

Considering that BCG is primarily administered in the clinic in newborn populations, we used a neonatal mouse model in which BCG was inoculated at birth, and MTBVAC HK given 8 weeks later, when the immune system has reached a mature status. Protection by Mtb reduction in lungs was significantly improved in the MTBVAC HK booster group (Figure 1D), and comparable to the protection level observed in adult mice immunized with BCG. We also evaluated vaccine efficacy by survival as a readout in a high-dose, mouse challenge model and found that intranasal MTBVAC HK boosting substantially extended mouse survival in comparison to BCG sc immunization (Figure 1E).

Although we did not investigate other administration routes, our results suggest that the beneficial effect of MTBVAC HK boosting specifically depends on its interaction with the respiratory mucosal immune system. Therefore we analyzed cellular responses in lungs as well as in spleen after *ex vivo* stimulation with *M. tuberculosis* secreted antigens (Purified protein derivative: PPD). Data revealed that MTBVAC HK intranasal boosting enhanced antigen-specific IFNγ and IL17 induction in lungs (Figure 1F). In addition, spleen response profiling also revealed a higher IFNγ and IL17 production at a systemic level elicited by MTBVAC HK boosting (Figure 1G).

### Intranasal MTBVAC HK Induces Protective Secretory Immunoglobulins

**FIGURE 2:**
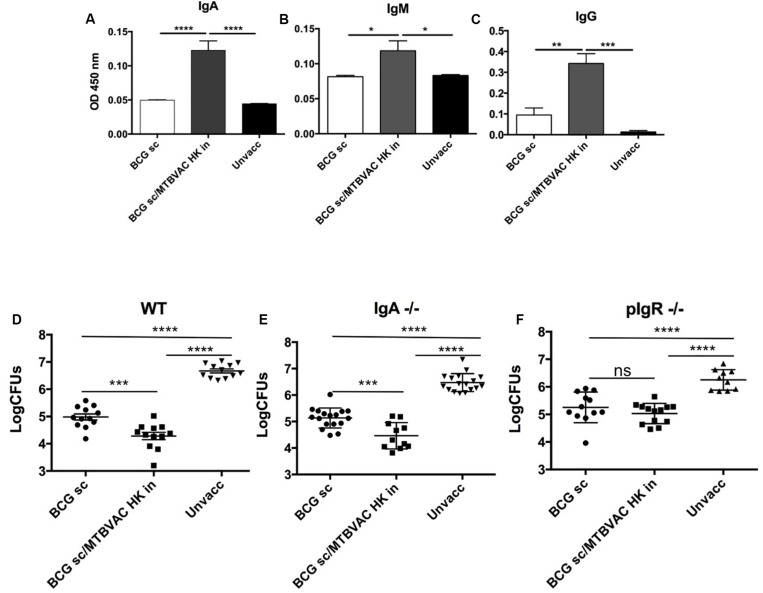
Intranasal MTBVAC HK induces mucosal immunoglobulins in respiratory airways. **(A–C)** Groups of C57BL/6 adult mice where vaccinated with BCG and MTBVAC HK 4 weeks apart. One month later PPD- specific IgA, IgM, IgG in BAL samples. **(D–F)** One month after MTBVAC HK boosting, wild-type, IgA-/- and pIgR-/- mice were intranasally challenged with H37Rv and lung bacterial load analyzed one month later. All data are mean ± SEM **(A–C)** Data are representative of two independent experiments (*n* = 6 mice/group). **(D–F)** Data in the graphs represent a pool of two independent experiments (*n* = 12 mice/group). *p < 0.05; **p < 0.01; ***p < 0.001; ****p < 0.0001 by one-way ANOVA and Bonferroni post-test.

We have previously reported that respiratory, but not subcutaneous live BCG, triggered IgA production in respiratory airways (Aguilo et al., 2016). Thus, we next assessed whether intranasal MTBVAC HK induced PPD-specific immunoglobulins, including IgA, IgM and IgG subtypes, in bronchoalveolar lavage (BAL) (Figures 2A–C). MTBVAC HK booster vaccination triggered an increase of the three types of antibodies compared to unvaccinated and BCG only control groups.

To assess contribution of mucosal secretory immunoglobulins (sIg) to MTBVAC HK-mediated protection, we tested vaccine-conferred protective efficacy in mice deficient by genetic knockout for IgA (igha-/-) or for the polymeric immunoglobulin receptor (pIgR-/-). This latter molecule is a protein transporter highly expressed in mucosal tissues, which binds to J chain to actively translocate multimeric IgA or IgM across mucosal epithelium, and therefore lack of pIgR leads to retention of multimeric sIg in the mucosal lamina propria, preventing their active transport over the mucosal barrier (Kaetzel et al., 1991). Our results revealed a similar protection provided by intranasal MTBVAC HK in igha-/- mice when compared to wild type (Figures 2D,E) suggesting no specific protective role of IgA. Conversely, MTBVAC HK-specific protection was completely abrogated in the absence of pIgR (Figure 2F). Altogether, this result would indicate a crucial contribution of sIg to protective efficacy mediated by MTBVAC HK, suggesting a substantial contribution of sIgM rather than sIgA. Remarkably, pIgR-/- mice did not show less protection by BCG s.c, suggesting that sIg has no role in systemic vaccine-induced protection.

### Mucosal MTBVAC-HK Booster Vaccination in NHP

**FIGURE 3:**
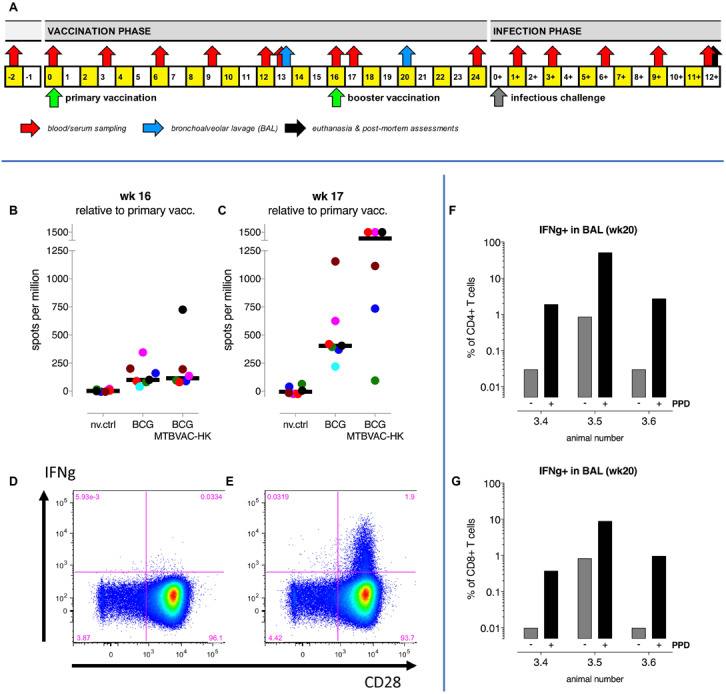
Intrabronchial MTBVAC HK boosting induces local and systemic tuberculosis-specific cellular responses in NHP. **(A)** A study design schematic shows the time lines on a weekly basis relative to primary BCG vaccination and infectious challenge with *M. tuberculosis*, including booster immunization and biosampling events. **(B,C)** PPD-specific IFNg responses in peripheral blood were measured by ELISPOT immediately prior to and 1 week after MTBVAC HK boosting, at week 16 and 17, respectively. Individual data points are consistently colored according to the Supplementary Table 1; fat horizontal lines indicate group medians. Also, flow cytometry after intracellular cytokine staining was used to analyze IFNg responses. Dot plots of CD28 versus IFNg specific fluorescence signals of CD4 + T lymphocytes from a representative BAL sample illustrate local IFNg production **(D)** in the absence and **(E)** in the presence of PPD recall stimulation *in vitro*. Percentages per individual of PPD-induced IFNg-positive **(F)** CD4+ and **(G)** CD8+T lymphocytes from BAL are depicted.

MTBVAC-HK was further evaluated as a booster vaccine in a non-human primate (NHP) vaccination and infection study to address tolerability, immunogenicity and protective efficacy in the primate host. Adult rhesus macaques (*Macaca mulatta*) were vaccinated either with BCG only, or with BCG followed by a MTBVAC-HK booster by pulmonary mucosal instillation 16 weeks later (Figure 3A). One group was left untreated as non-vaccinated controls. Twenty-five weeks after primary vaccination all animals were challenged by endobronchial instillation of 50 colony forming units (CFU) of *Mtb* strain Erdman, with a follow-up of 12 weeks until study endpoint and post-mortem evaluation of infection and disease (Figure 3A). (See Supplementary Table 1 for an overview of animals per treatment group).

Within the limits of observation, animals tolerated mucosal MTBVAC-HK boosting well; we did not observe any adverse events. Measuring vaccine-induced responses by specific interferon-gamma (IFNg) ELISPOT, using PBMC and antigenic recall stimulation with PPD, MTBVAC-HK boosting induced a transient elevation of the frequency of IFNg producing cells in the periphery at week 17 (one week post MTBVAC HK immunization) relative to the response induced by BCG (*p* = 0.0997 by non-parametric Mann-Whitney testing; Figures 3B,C). This increase was found to be transient since MTBVAC HK-specific T cell response dropped sharply at week 20 (Supplementary Figure S5A). Locally, and examining three pre-assigned animals out of six of the MTBVAC-HK boosted animals only (comparing week 13 versus week 20 post-BCG), we registered a clear influx of cells into the lung lumen upon mucosal MTBVAC-HK boosting and the induction of IFNg-positive CD4+ and CD8+ T lymphocytes (Figures 3D–G).

**FIGURE 4:**
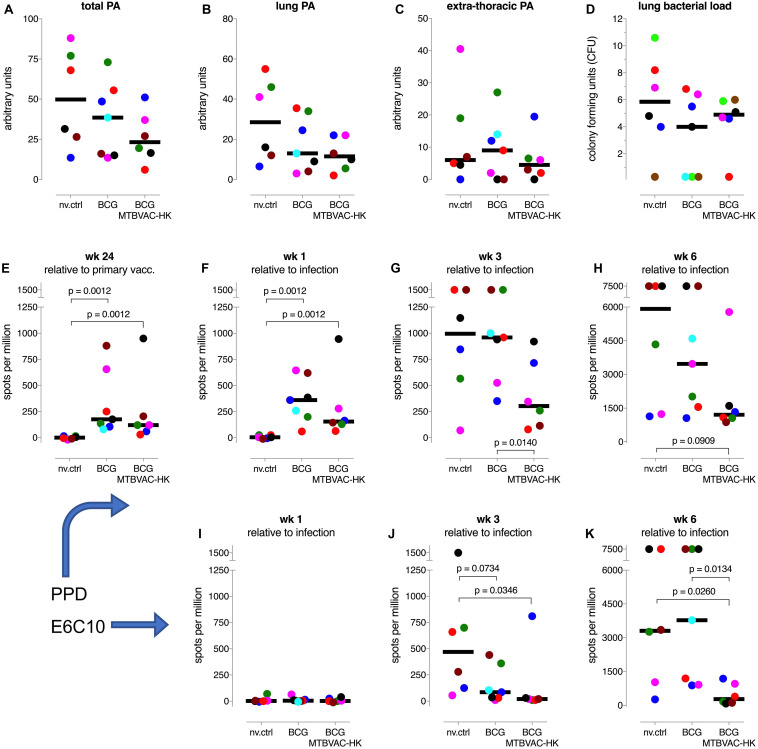
TB disease measures and IFNg responses after infectious challenge. **(A–C)** Total arbitrary score of pathological involvement at endpoint and for lung and extra-thoracic disseminated disease, respectively. **(D)** Post-mortem enumeration of bacterial burden in the lung. **(E–H)** PPD-specific and **(I–K)** ESAT6-CFP10 fusion protein (E6C10) specific IFNg responses by specific ELISPOT analysis of PBMC, at indicated timepoints relative to primary vaccination or infectious challenge. Individual data points are consistently colored according to the Supplementary Table 1; fat horizontal lines indicate group medians.

Upon infectious challenge with *Mtb*, highest pathology scores and lung bacterial counts were obtained in 3 non-vaccinated control (nv.ctrl) animals (Figures 4A–D, and Supplementary Figures S5B,C). However, there was an unexpected large spread in pathological involvement, with three out of six unvaccinated controls showing relatively mild disease levels (for which we have no explanation at this point), which altered all the statistical comparisons. Both BCG vaccinees and the MTBVAC-HK boosted animals revealed lower pathology scores by group median values (except for extrathoracic dissemination), but without statistical significance (Figures 4A–C). Likewise, there was no statistically significant vaccine effect by enumeration of *Mtb* at necropsy from lung (Figure 4D) or hilar lymph node and spleen (Supplementary Figures 4B,C, respectively). Despite the lack of statistically significant improvement, by various parameters there appears some positive trend by group median scores of improved TB disease outcome after MTBVAC-HK boosting relative to BCG alone: for total pathology score (Figure 4A), body weight development, and infection-associated anemia by hematological mean corpuscular hemoglobin (MCH) and mean corpuscular volume (MCV) development (Supplementary Figures S5D,F,G, respectively).

Most notably, MTBVAC-HK boosting significantly suppressed the PPD-specific IFNg response up until 6 weeks after infection (Figures 4E–H). Also, the *Mtb*-specific response against an ESAT6-CFP10 fusion protein (covering antigens which are absent from *M. bovis*-derived BCG vaccine) is significantly suppressed up until 6 weeks post-infection by MTBVAC-HK boosting (Figures 4I–K). While we have recently established the correlation between lower anti-*Mtb* IFNg responses following challenge and protective immunity against infection and disease in a repeated limiting dose (RLD) infection model in rhesus macaques (Dijkman et al., 2019), we consider that these unprecedented, suppressed PPD and ESAT6-CFP10 specific responses might reflect the protective effect of MTBVAC-HK boosting in this NHP model under relatively high-dose challenge condition. Most likely because of the relatively high challenge dose of 50 CFU of *Mtb*, we anticipate that, from 6 weeks post-infection onward, disease in these highly susceptible rhesus macaques can no longer be controlled. As a consequence, pathology scores and mycobacterial counts at endpoint may have converged to a similar outcome leaving a trend of improvement by MTBVAC-HK boosting only.

### MTBVAC HK Vaccination Induces Mucosal Immunoglobulins in Vaccinated NHP

**FIGURE 5:**
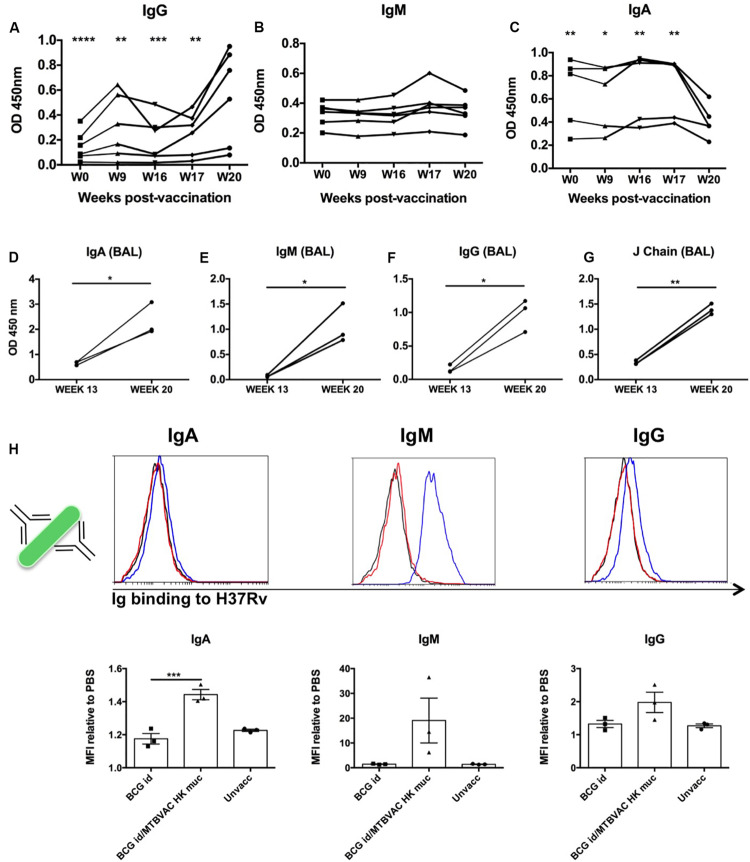
Intrabronchial MTBVAC HK induces mucosal immunoglobulins in respiratory airways from non-human primates. **(A–C)** PPD-specific IgG, IgM, and IgA were measured in sera samples from the different individuals throughout the vaccination phase (until week 20). **(D–G)** PPD- specific IgA, IgM, IgG, as well as J chain were analyzed in BAL samples harvested at week 13 (before MTBVAC HK) and week 20 (after MTBAVC HK) from three individuals from the MTBVAC HK group. Data in the graphs show values for each individual **(H)** Direct binding of IgA, IgM, and IgG to Mtb surface. H37Rv bacteria were incubated with BAL samples and immunoglobulin binding measured by flow cytometry using specific secondary antibodies. Representative overlay histograms are shown. Black line: unvaccinated; Red line: BCG vaccinated; Blue line: BCG/MTBVAC HK vaccinated. Data in the graphs show the mean fluorescence intensity (MFI) obtained with each BAL compared to the measured when bacteria are incubated with PBS. **(A–G)** Data show individual from one experiment. **(F)** Data are shown as mean ± SEM and are representative of two independent experiments. **(A–C, H)** *p < 0.05; **p < 0.01; ***p < 0.001; ****p < 0.0001 by one-way ANOVA and Bonferroni post-test. **(A–C)** Comparisons of the different timepoints with week 20 are shown. **(D–G)** *p < 0.05; **p < 0.01 by paired *t*-student test.

MTBVAC HK mucosal vaccination induced a strong cellular response both systemically and locally. However, considering the results obtained in mice we focused further analyses on systemic and local antibodies. To this end, we used sera and BAL samples from vaccinated monkeys obtained along the study time line to characterize humoral immune responses. PPD-specific IgG, IgM and IgA kinetics were monitored in serum samples from MTBVAC HK group during vaccination phase (from week 0 to week 20). Specific IgG showed a significant increase at week 20, which was particularly pronounced in 4 of the 6 animals, indicating a correlation between PPD-specific IgG and MTBVAC HK vaccination (Figure 5A). In the case of IgM, no significative changes were observed at the timepoints analyzed, although a slight transitory increase was observed in two animals at week 17, one week after MTBVAC HK inoculation (Figure 5B). Finally, the PPD-specific IgA profile showed a decrease that correlated with the administration of MTBVAC HK (Figure 5C).

We obtained BAL samples of three monkeys from the MTBVAC HK group at two different timepoints: at week 13 (before MTBVAC HK booster immunization), and at week 20 (4 weeks after MTBVAC HK administration). Our results clearly indicate that mucosal vaccination with MTBVAC HK induced a substantial increment of PPD-specific IgA, IgG, and IgM in the three animals studied, similar to the observations in mice. In addition, we also observed an increase in J chain presence, suggesting the presence of multimeric secretory IgA and IgM (Figures 5D–G).

Our data demonstrate the induction of PPD-specific immunoglobulins by MTBVAC HK. However, PPD comprises the secreted fraction of *M. tuberculosis* proteome. Thus, we next aimed to elucidate whether MTBVAC HK-induced mucosal Ig could bind to *M. tuberculosis* bacilli. To this end, we incubated H37Rv bacteria with BAL fluid samples, followed by biotin-conjugated secondary antibodies specific for human IgM, IgG and IgA. A final incubation step with PE-bound streptavidin allowed visualization of Ig binding by flow cytometry. Our data show a positive shift of the fluorescence signal for either of the three immunoglobulin families, IgA, IgM, and IgG, when bacteria had been incubated with BAL fluid from MTBVAC HK-vaccinated animals, indicating the presence of IgA, IgM, and IgG, able to bind to Mtb directly. This binding was substantially higher in the case of IgM, which would correlate with the pentameric conformation of this immunoglobulin. While for the BCG only-vaccinated animals, the binding was similar to that measured in BAL samples from unvaccinated animals, the highest signals were obtained after MTBVAC HK boosting specifically (Figure 5H).

### H37Rv Opsonization Following Incubation With MTBVAC HK-Derived BAL

**FIGURE 6:**
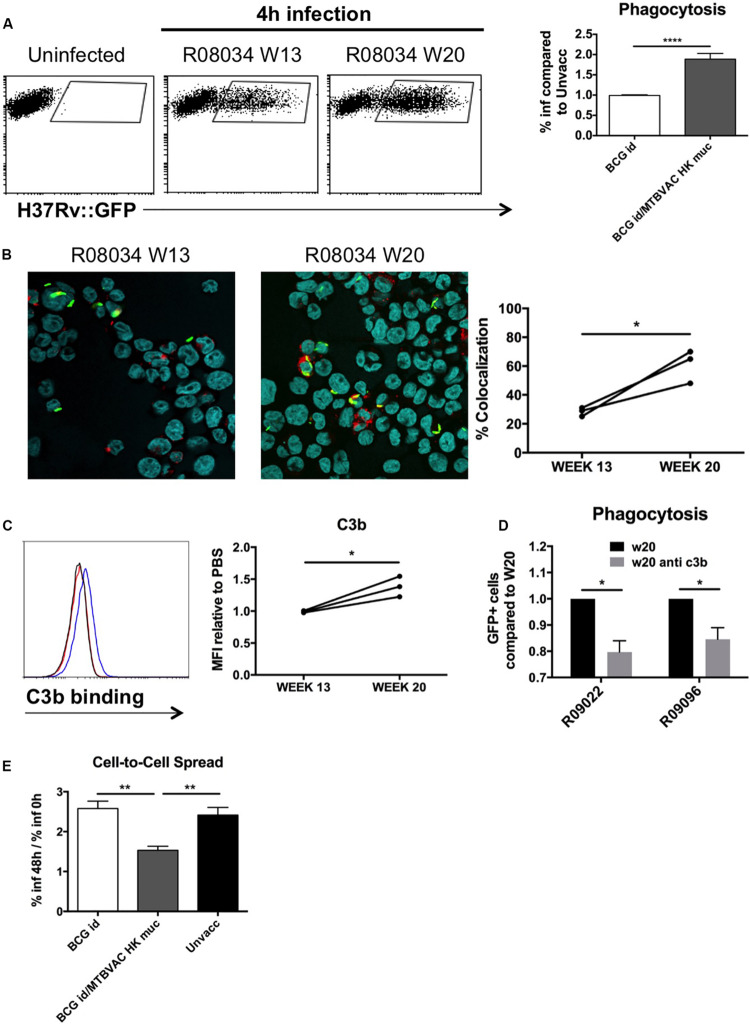
Mucosal immunoglobulins induced by MTBVAC HK opsonize Mtb *in vitro*. GFP-expressing H37Rv coated with BAL samples were added to THP-1 human monocytes. **(A)** Percentage of infected cells was determined by flow cytometry 4 h post-infection. Representative dot-plots from one individual are shown in the left panel. Data in the graph is represented as the fold-change of the percentage obtained with the BAL from BCG-only or BCG/MTBVAC HK compared with the BAL from unvaccinated NHP. **(B)** Representative images of infected cells stained with lysotracker and Hoechst reagents. Data in the graph show colocalization values for each individual from MTBVAC HK group comparing colocalization obtained with BAL from week 13 and 20. **(C)** C3b binding to H37Rv surface was analyzed by flow cytometry using an antibody against C3b. A representative overlay histogram is shown. Black line: unvaccinated; Red line: BCG vaccinated; Blue line: BCG/MTBVAC HK vaccinated. Data in the graph show the MFI fold-change obtained following incubation with BAL from week 13 and 20 compared to the measured with the value when bacteria are incubated with PBS. **(D)** THP1 cells were infected with H37Rv previously incubated with BAL from week 20 in the presence or absence of a neutralizing antibody antiC3b. **(E)** Fold-change comparison of infected- THP1 cells at 48 h versus 0 h for each experimental condition. **(A,E)** Data in the graphs are mean ± SEM from a pool of three independent experiments. **p < 0.01; ****p < 0.0001 by one-way ANOVA and Bonferroni post-test. **(B)** Data show mean values of at least six images for each experimental condition from one experiment. **(C)** Data show individual from one experiment. **(B,C)** *p < 0.05 by paired *t*-student test. **(D)** Data in the graphs are mean ± SEM from a pool of two independent experiments. *p < 0.05 by two-way ANOVA and Sidak’s multiple comparison test.

Phagocytosis mediated by antibody-dependant opsonization represents a major bactericidal mechanism triggered by immunoglobulins. To evaluate opsonization in our study, we added H37Rv to human monocyte cells THP1, after it had been incubated with the different BAL samples. We used GFP-expressing bacteria in order to monitor internalization by flow cytometry. Our data demonstrate about a two-fold increase of infected cells when H37Rv had been previously incubated with BAL fluid from MTBVAC HK-vaccinated monkeys compared to control groups (Figure 6A). Opsonization has been associated with an increase in the capacity of macrophages to restrict bacteria into acidic compartments. To assess this, we analyzed intracellular colocalization of H37Rv with lysotracker, a probe that becomes fluorescent under acidic conditions. In the three monkeys analyzed, our results showed an increased bacterial colocalization following pre incubation of H37Rv with BAL from week 20 compared to those from week 13 (Figure 6B).

Our results showed great ability of BAL IgM and IgG to bind Mtb surface. Complement activation in respiratory airways has been previously reported, and therefore, since IgM and IgG is a very efficient complement activators, we evaluated the role of C3b, one of the major opsonins released during complement activation, for MTBVAC HK vaccination-induced opsonization. We incubated H37Rv with the different BAL samples and then we added a C3b-specific antibody to detect binding of this protein to Mtb surface by flow cytometry. In line with what was observed for the different immunoglobulin subtypes previously, we detected a higher fluorescence peak of C3b binding with the BAL from week 20 compared to week 13 (Figure 6C). Interestingly, preincubation of week 20 BAL from two different individuals with anti-C3b partially prevented Mtb phagocytosis, suggesting a functional role of this opsonin in enhanced uptake (Figure 6D). These results do not rule out that other opsonins released during complement activation play a role in the enhanced uptake of Mtb after MTBVAC HK booster vaccination.

Finally, we analyzed the capacity of internalized bacteria to spread from cell to cell. Our data indicate that percentage of infected cells at 48 h of infection was about 2.5-fold higher when compared to initial infection rate in cases where H37Rv had been incubated with control BAL fluid (unvaccinated or BCG only), whereas this ratio dropped to 1.5 with the BAL from MTBVAC HK boosted animals (Figure 6E). This result suggests that the ability of Mtb to spread to bystander cells was impaired when bacteria were opsonized.

## Discussion

During the last years there is rising interest to elucidate the role of immunoglobulins in tuberculosis infection. Different studies using animal models have shown that pulmonary delivery of monoclonal antibodies targeting certain Mtb surface proteins leads to a reduction of bacterial load following challenge (Achkar and Casadevall, 2013). In addition, results from clinical vaccine trials suggest a correlation between Mtb-specific IgG in sera and lower risk of TB disease (Fletcher et al., 2016). An elegant study recently demonstrated differences in glycosylation of immunoglobulin Fc fractions between latent and active TB individuals, and the importance of these signatures for antibody interaction with different cellular subsets, suggesting a role of antibodies in natural prevention of TB reactivation (Lu et al., 2016). Altogether, these studies provide substantial support for the potential contribution of antibodies to natural or vaccine-induced protection against tuberculosis.

Nevertheless, there is a poor understanding about the processes that drive to generation of protective antibodies during tuberculosis infection, and vaccine strategies that specifically exploit humoral responses are underrepresented in the tuberculosis vaccine pipeline. In the present work, we identify a novel vaccine approach that induces mucosal protective antibodies in the lungs. We describe in mice how a single intranasal immunization with a inactivated whole-cell *M. tuberculosis* vaccine triggers lung protective immunity when given as boost over previously BCG immunized animals. Data from NHP studies also showed a tendency in the MTBVAC HK-immunized group to improve protection in certain disease-associated parameters, even though differences were not statistically significant. Remarkably, results in NHP showing MTBVAC HK-induced inhibition of PPD- and ESAT6/CFP10-specific responses following Mtb challenge suggest a protective role of MTBVAC HK in this animal model. However, high dose challenge experimental conditions used in this study may be obscuring the full protective potential of MTBVAC HK boosting.

We show that mucosal administration of MTBVAC HK induces strong cellular and humoral responses at both systemic and local lung level, which correlates with an improved protection compared to animals immunized only with BCG by parenteral route. Our data evidence the importance of the mucosal route of administration to trigger mucosal immunoglobulins in the lungs. In this regard, we demonstrate that intradermal BCG, the current vaccine regimen used in the clinic, does not generate antibodies in the lung lumen, which is in agreement with our previous observations (Aguilo et al., 2016).

Remarkably, MTBVAC HK fails to protect in the absence of pIgR, a transporter protein that mediates release of secretory Ig (sIg) to respiratory airways, suggesting a crucial role of mucosal antibodies in the protective mechanism triggered by this vaccination strategy. sIg comprise polymeric IgA (dimer) or IgM (pentamer) molecules, covalently linked to J chain, and the secretory component (SC), which corresponds to a pIgR extracellular domain that is cleaved following translocation. This complex conformation provides more stability to the immunoglobulin, making it less susceptible to proteolytic digestion and more mucophilic (Rojas and Apodaca, 2002). Our results in mice suggest no contribution of IgGs to MTBVAC HK-mediated protection, despite their presence in BAL following immunization. Monomeric immunoglobulins have lower capacity to neutralize pathogens in mucosal tissues compared to polymeric conformations (Renegar et al., 1998). In this regard, our results suggest a strong capacity of sIgA and sIgM to impair *M. tuberculosis* infection.

Even though our data do not demonstrate greater susceptibility of IgA-/- and pIgR-/- mice to Mtb infection *per se* and therefore, no apparent role of sIg in natural TB protection, the scenario changes when Mtb-specific sIg are present in the respiratory airways prior to pathogen encounter. Our *in vitro* functional assays using BAL fluid samples from MTBVAC HK-vaccinated NHP indicate the capacity of vaccine-induced sIg to bind directly to Mtb surface and to modulate pathogen interaction with macrophages. Mtb incubation with BAL from MTBVAC HK-immunized monkeys led to an increase of the phagocytosis rate, a higher colocalization of intracellular Mtb with acidic compartment tracer, and an impaired capacity of bacteria to spread from cell to cell. Thus, our data suggest antibody-mediated opsonization as a plausible protective mechanism of MTBVAC HK vaccination.

Our results indicate similar MTBVAC HK-induced protection in IgA-/- mice (where IgM is the only secretory immunoglobulin available) compared to wild-type animals, suggesting a primordial role of sIgM in vaccine-induced protective efficacy. However, our results are not sufficient to determine that IgA have not contribution to vaccine-induced protective response. A good approach to elucidate this point could be the use of a mouse strain unable to produce secretory IgM. This experiment should be performed in the future to clarify this question. Unlike IgG and IgA, it is not well defined up-to-date which receptor for IgM could drive IgM-specific opsonization. However, IgM, including secretory IgM (Michaelsen et al., 2017), is a strong complement activator, and IgM-dependent opsonization by complement-derived opsonins is well reported (Guidry et al., 1993). In addition, our results also revealed an important binding of IgG to *Mtb* surface. Since IgG is a strong complement activator, these antibodies might also play a role in the bacterial opsonization events described in this study. One of the major opsonins activated during this process is C3b, released as a consequence of the C3 proteolization by the enzyme C3-convertase. C3b presents strong avidity of binding to bacterial surfaces directly, and it mediates opsonization following recognition by CD35, a surface receptor on macrophages, including the THP1 cells used in the present study (Baqui et al., 1999). Our results suggest that, at least partially, C3b generated following MTBVAC HK vaccination has a role in Mtb opsonization. Although complement proteins are mostly generated in the liver, lungs can be a local source of complement proteins. In particular, alveolar epithelial cells, as well as alveolar macrophages can secrete complement proteins (Strunk et al., 1988; Huber-Lang et al., 2002).

Despite the lower immunogenicity and poorer protective efficacy conferred by inactivated whole-cell tuberculosis vaccines in comparison to their live counterparts, there is an interest in the development of this type of vaccination strategies. As inactivation is associated with an improved safety profile, such vaccines are considered in particular for target populations to which live vaccines might be harmful, such as immunosuppressed individuals. Limited immunogenicity of inactivated vaccines is usually overcome by other strategies, such as the formulation of inactivated vaccines with adjuvants (Haile et al., 2004) or repeated administration by multiple immunizations (Opie and Freund, 1937; Von Reyn et al., 2017). In the case of intranasal MTBVAC HK, our data indicate that it is not protective by itself, but it needs of a previous BCG prime to trigger a response in the lungs and confer protection. Even though we have not fully studied the mechanisms behind this result, it could be interesting to address the cross-talk between mucosal and systemic immune compartments in the future. For now, we speculate that the mucosal delivery of MTBVAC HK might be pulling the systemic cellular response elicited by subcutaneous BCG toward the lungs. Secondary targeting of the lung compartment with inactivated bacilli, would be a relatively safe way of boosting the cellular response and inducing specific antibodies locally against whole-cell bacteria.

Our results do not clarify whether the present vaccination approach is effective only when we use an *M. tuberculosis*-based vaccine as MTBVAC, or whether it can be translated to other whole-cell vaccines like inactivated BCG. In this regard, a previous study reported no additional protection in the lungs conferred by intranasal heat-killed BCG given as booster of subcutaneous live BCG immunization (Haile et al., 2005). This failure of boosting with inactivated BCG could highlight the importance of the differential antigens expressed by MTBVAC relative to BCG (Marinova et al., 2017), which are shared with the *M. tuberculosis* pathogen. Indeed, contribution of differential antigens to MTBVAC-conferred protection has been previously reported in the case of live MTBVAC (Aguilo et al., 2017). Nevertheless, comparison between inactivated MTBVAC and BCG should be done head-to-head in order to confirm this difference under the same experimental conditions.

The results from the present study indicate that a plausible mechanism of protection of mucosal MTBVAC HK is mediated by the generation of specific antibodies in the respiratory airways, with capacity to opsonize live Mtb bacteria. We do not discard that another inflammatory events mediated by the vaccine as production of certain cytokines could also contribute to protection. To our knowledge, this is the first tuberculosis vaccine approach based on whole-cell bacteria that targets to elicit protective mucosal antibodies.

## Materials and Methods

### Bacteria

BCG Danish (Statens Serum Institute) and H37Rv (Institut Pasteur Paris) strains were grown at 37°C in Middlebrook 7H9 broth (Difco) supplemented with ADC 10% (Difco) and 0.05% (v/v) Tween-80 (Sigma), or on solid Middlebrook 7H11 (Difco) supplemented with ADC 10%. Bacterial suspensions for vaccination or infection were prepared in PBS from glycerol stocks previously quantified by plating serial dilutions. MTBVAC HK vaccines were prepared by heating a log-phase MTBVAC culture at 100°C during 30 min. In the case of formalin inactivation, MTBVAC culture was incubated with 0.5% (vol/vol) formalin during 48 h. Culture viability was checked for each vaccine lot. Bacterial density was determined by plating prior to heat treatment, and was used to calculate MTBVAC HK dose for *in vivo* studies.

### Cell Lines

THP-1 cells were purchased to European Collection of Cell Cultures (ECACC) (Lot Number 10/016). All the experiments were done using cells with less than five passages from its thawing. Mycoplasma absence in the cultured cells was confirmed after finalizing the experiments using the MycoAlert PLUS Mycoplasma Detection Kit (Lonza).

### Animal Studies Approval

Mouse studies were conducted in agreement with European and national directives for protection of experimental animals and with approval from the Ethics Committee from University of Zaragoza (approved protocols PI14/14, PI50/14, and PI33/15).

Ethical approval for NHP study was obtained from the Dutch authorities prior to start, under dossier registration number DEC726subB.

### Mouse Study

All mice were kept under controlled conditions and observed for any sign of disease.

#### Protective Efficacy

Female, 8–10 weeks-old C57BL/6 (Janvier Biolabs), pIgR-/- (a kind gift from Gerard Eberl, Institut Pasteur Paris), IgA-/- (MMRRC repository), or DBA/2J (Janvier Biolabs) mice were vaccinated subcutaneously with 5 × 10^5^ CFU BCG vaccine in 100 μl PBS. Four weeks post-vaccination, mice were intranasally vaccinated with 10^7^ MTBVAC HK bacteria in 40 μl of PBS. Four weeks later, mice were challenged intranasally with 150 CFUs H37Rv. Bacterial burden was assessed four weeks post-challenge by plating homogenized lungs and spleen on solid medium. A group of infected mice was sacrificed 1 day after challenge in order to determine the initial bacterial load in lungs, which resulted in approximately 20 CFU in all experiments. In the experiments involving newborn animals, mice were vaccinated subcutaneously with 2.5 × 10^5^ BCG CFU in 50 μl PBS in the first three days after birth. Unvaccinated controls were inoculated with 50 μl of PBS. 10^7^ MTBVAC HK was administered intranasally eight weeks later (or 10^4^ when indicated). Four weeks later, mice were challenged with 150 CFUs of H37Rv for bacterial burden determination, or 10^4^ CFUs for survival evaluation. In this latter case, disease-associated symptoms (including weight, aspect and individual/social behavior) were monitored weekly, and mice were humanely euthanized according to pre-established endpoint criteria.

#### Immunogenicity

Four weeks after MTBVAC HK vaccination, mice were euthanized and splenocytes and lung cells collected. 10^6^ cells per experimental timepoint were stimulated with Purified Protein Derivative (PPD) (Statens Serum Institute, SSI) 5 μg/ml during 48 h for supernatants collection and cytokine detection by ELISA. IL17A or IFNγ concentrations were determined with specific ELISA commercial kits (MabTECH). For bronchoalveolar lavage (BAL) collection, trachea was cannulated and BAL was performed with 0.8 ml of ice-cold PBS. Supernatant was separated from cells by centrifugation and frozen at −80°C for further protein detection analysis. For antibodies or J chain determination in BAL, maxisorp ELISA plates (NUNC) were coated with 10 μg/ml of PPD and incubated overnight at 4°C. After a washing step with PBS-Tween20 0.05% (v/v) buffer, plate was blocked with Bovine Serum Albumin 1% (w/v) in washing buffer for 1 h at 37°C. Then, plates were incubated with 100 μl of BAL during 90 min at 37°C. Following washing, plates were incubated for 1 h at 37°C with the corresponding anti IgA, IgG, or IgM antibodies conjugated with Horseradish Peroxidase (HRP) (Sigma Aldrich) at a 1:10000 dilution. Finally, enzyme-substrate reaction was developed using 3,3′,5,5′-Tetramethylbenzidine (TMB) (Sigma Aldrich) as substrate, and reaction was stopped with H_2_SO_4_ 0.1N.

### NHP Study

#### Bacteria, Vaccination and Infectious Challenge

For primary vaccination of NHPs, clinical grade BCG Danish 1331 (SSI, Copenhagen, Denmark) was reconstituted according to manufacturer’s protocol and applied at a single, standard human dose (of 5 × 10^5^ CFU on average) by intradermal injection.

Inactivated MTBVAC-HK booster vaccination in NHP was established by endobronchial instillation of 1.95 × 10^8^ CFU-equivalent/10 mL/dose, targeting the lower left lung lobe. To this end, one mL of MTBVAC-HK suspension (provided by Biofabri) was added to 9 mL of sterile, isotonic saline solution (Eurovet Animal Health B.V., Bladel, Netherlands) immediately prior to administration.

*M. tuberculosis* (Mtb) strain Erdman K01 was prepared at 50 colony forming units (CFU) per 3 mL sterile, isotonic saline per dose, for infectious challenge of NHPs by endobronchial instillation targeting the lower left lung lobe. NHPs were challenged in random order in a single session within 3 h from the preparation of the inoculum, using a pool of 3 vials of Mtb Erdman from −80°C frozen stock.

#### Study Design, Handling and Sampling

Purpose-bred, adult, healthy, Indian-genotype rhesus macaques (Macaca mulatta) were selected from BPRC’s breeding colonies and stratified into treatment groups. Males and females were represented at a 2:1 ratio per group (unless indicated otherwise; see Supplementary Table 1). Animals, per gender, were socially housed throughout the experiment in BPRC’s experimental animal facilities at biosafety level three. Enrichment was provided in the form of food and non-food items and welfare was monitored by daily observation.

A schematic diagram of the study design is provided in Figure 3A. After a brief period of acclimatization and baseline measurements, animals were primed with BCG – or left untreated as non-vaccinated controls – and BCG vaccinees were either or not boosted with MTBVAC-HK 16 weeks later. Another 9 weeks later – that is 25 weeks post-priming – all animals received an infectious challenge with 50 CFU of Mtb Erdman into the lower left lung lobe. Twelve weeks post-infection, animals were sedated and euthanized by intravenous pentobarbital injection, and subsequently submitted to full post-mortem evaluation and assessment of pathological and bacteriological parameters of infection and disease. Two animals of the non-vaccinated control group reached a premature, humane endpoint due to acute progressive disease development and were euthanized for full post-mortem evaluation 6 and 11 weeks post-infection, respectively (see Supplementary Table 1).

All animal handling was performed under ketamine sedation (10 mg/kg, by intra-muscular injection). For pulmonary endoscopic installation and additional relaxation, intramuscular ketamine (5 mg/kg) was supplemented with medetomidine (0.04 mg/kg) and an analgesic was sprayed into the larynx.

Peripheral blood was sampled by venepuncture at various time points, as indicated in the Results section. For immunological assays, peripheral blood mononuclear cells (PBMC) were isolated from heparinized blood by standard Ficoll^®^-based gradient centrifugation using Lymphoprep^TM^ (Axis-Shield, United Kingdom). Serum tubes were spun for 10 min at 1000 g to harvest and store cell-free serum at −80°C for further analysis at later time point. Serum samples were filter-sterilized using 0.2 μm PVDF-membrane plates (Fisher Scientific) before immune response analysis.

Standard hematological parameters were measured in fresh EDTA blood on a Sysmex2000i system (Siemens). Standard clinical chemistry values were determined in fresh serum samples on a Cobas^TM^ Intregra 400+ platform (Roche Diagnostics).

Bronchoalveolar lavage (BAL) was collected by three times consecutive instillation-and-harvesting of 20 mL prewarmed, sterile 0.9% saline, targeting the lower left lung lobe with an endoscope. BAL was collected 3 weeks before and 4 weeks after MTBVAC-HK boosting from *N* = 3 pre-assigned animals of the MTBVAC-HK booster group, and 3 weeks before boosting also from *N* = 3 pre-assigned animals of the non-vaccinated controls (see also Supplementary Table 1 and Figure 3A). After passage over a 100 μm filter and centrifugation for 10 min at 400 g, supernatant was decanted and stored as BAL fluid at −80°C pending further analysis. BAL cell pellet was resuspended in fetal calf serum-, glutamax- and penicillin/streptomycin-supplemented RPMI culture medium for immediate use in immune assays. BAL fluid samples were filter-sterilized using 0.2 μm PVDF-membrane plates (Fisher Scientific) before immune response analysis.

#### NHP Post-mortem Evaluation

At study endpoint, gross pathology was arbitrarily scored utilizing a predefined algorithm, adapted from Lin et al. (2009). Lung lobes were slabbed into approximately 0.5 cm slices for ample inspection and lesions were scored by size, frequency and appearance. Extrathoracic organs (typically spleen, liver and kidneys) were scored similarly, and lymph node involvement was graded by size and appearance.

After gross pathological examination, lung lobes were minced and spleen and lung-draining lymph nodes were passed over a 100 μm cell strainer (Greiner Bio One International), prior to further homogenization using GentleMACS M tubes (Miltenyi Biotec). Homogenates were frozen stored at -80°C and later thawed and serially diluted onto Middlebrook 7H10 plates for enumeration of bacterial tissue burden.

#### NHP Immune Analyses

Immunogenicity of vaccination was measured by a lymphocyte stimulation test using a specific NHP IFNg ELISPOT kit (U-CyTech) for the readout of the frequency of IFNg producing cells. In brief, 2 × 10^5^ PBMC in supplemented RPMI culture medium were incubated in triplicate in microtiter flat-bottom plates for 24 h at 37°C and 5% CO2, either or not in the presence of specific antigen. Mtb-derived protein purified derivative (PPD, Statens Serum Institute, Denmark) or Mtb-specific, recombinant ESAT6-CFP10 fusion protein (E6C10, for short; a gift from K. Franken, LUMC, Leiden) were used for *in vitro* recall stimulation as indicated, both at a final concentration of 5 μg/mL. After overnight incubation cells were transferred to specific anti-IFNg-coated filter plates (PVDF, Millipore) for an additional overnight incubation. Spots were developed using tetra-methylbenzidine substrate (MAbTech) and quantified on an AELVIS automated reader.

Flow cytometry was used to measure intracellular IFNg cytokine levels in CD4+ and CD8+ T lymphocytes from BAL. In brief, BAL cells (at least 1 × 10^6^ cells) were either or not stimulated with PPD at a final concentration of 5 μg/mL in supplemented RPMI for 3 h in the presence of costimulatory antibodies against CD28 (PE-Cy7 conjugated) and CD49d. Subsequently, Golgiplug (BD Biosciences) was added and cells were incubated for another 15 h at 37°C and 5% CO2. Cells were then stained by standard procedures using Cytofix/Cytoperm and PermWash buffer (all from BD Bioscience), using the following antibody conjugates: anti-CD20-V450, anti-CD3-V500, anti-CD4-PerCP-Cy5.5, anti-CD8a-APC-Cy7, and anti-IFNg-AF700. T lymphocytes were gated as singlets, CD20-negative, CD3-positive cells. Within this population CD4+ and CD8+ cells were interogated for cytokine production (Supplementary Figure S6). Cells were measured in a LSR-II flowcytometer (BD Biosciences); data were analyzed using FlowJo software.

PPD-specific IgA, IgM and IgG in sera and BAL samples, as well as J chain in BAL, were determined following a protocol similar to the one described above for mice. For J chain determination, plates were incubated with a primary monoclonal antibody anti J chain (Santa Cruz Biotechnology, sc-271967) at a 1:1000 dilution, followed by incubation with secondary anti mouse IgG. Serum samples were diluted 1:500 in all cases, whereas BAL fluid was used undiluted. Anti human IgA, IgG or IgM antibodies were all conjugated with HRP (Sigma Aldrich) and diluted 1:10000. Primary anti J chain antibody was the same as described above for mice.

#### Functional Studies With BAL Samples

To evaluate direct Ig binding to Mtb, 10^7^ GFP-expressing H37Rv in 50 μl of PBS were added to 50 μl of BAL (or PBS as control) and incubated for 1 h at room temperature. Then, anti human IgA, IgG or IgM antibodies, all biotin-conjugated (Mabtech), were added in 50 μl at a final dilution of 1:200 (30 min at room temperature). Finally, streptavidin conjugated with APC (Miltenyi Biotech) was added in 50 μl at a final dilution of 1 μg/ml (30 min at room temperature). Bacteria were fixed adding 300 additional μl of paraformaldehyde (PFA) (final concentration 4%) and acquired with a flow cytometry Gallios (Beckman Coulter). Use of GFP-expressing H37Rv allowed discerning bacteria from BAL-derived debris. To assess C3b binding, we used an anti C3b as primary antibody (Millipore, clone 3E7) diluted at 1:25. Biotin-conjugated anti mouse IgG (Bethyl), diluted 1:200, and APC-conjugated streptavidin were used to detect fluorescence by flow cytometry.

THP-1 cells (Sigma-Aldrich) were cultured at 37°C and 5% CO_2_ in DMEM medium (Invitrogen) supplemented with 10% inactivated foetal bovine serum (Biological industries) and 2 mM glutamine (Biological industries). 5 × 10^5^ cells were seeded in 24-plate wells and incubated overnight with PMA 10 ng/ml. GFP-expressing H37Rv were incubated with the different BAL samples (or PBS as control) as described above. When indicated, anti C3b at 300 μg/ml was added to BAL suspensions. Then, bacterial suspensions were prepared in DMEM complete medium and put in contact with cells for 4 h at a MOI or five bacteria per cell. Cells were washed three times with PBS and detached with trypsin to evaluate internalization by flow cytometry. In some of the experiments, fresh media was added and GFP-positive cells analyzed 48 h later. To assess bacterial colocalization with acid compartments, cells seeded over microscopy slides were incubated with 1 μM lysotracker red (Invitrogen). Then, they were fixed with PFA and nuclei stained with Hoechst 33342 10 μg/ml. Images were acquired with an Olympus FV10-i confocal microscopy. Colocalization was quantified using FV10-ASW 3.0 software (Olympus), and was calculated as the proportion of pixels that were both green and red compared to those that were green. Percentage of colocalization was determined analysing at least six images randomly taken for each experimental condition.

### Statistical Analysis

Results from this study were not blinded for analysis. No randomization specific methodology was applied to this study. No statistical method was used to calculate sample size in animal experiments. GraphPad Prism six software was used for statistical analysis. Statistical tests used for each experiment are indicated in the figure legends. All statistical tests were two-tailed. Outlier values were determined applying the Grubb’s test to all data sets, and were discarded from the final statistical analysis. Differences were considered significant at *p* < 0.05.

## Data Availability Statement

The datasets generated for this study are available on request to the corresponding author.

## Ethics Statement

The animal study was reviewed and approved by Comisión Ética de Experimentación Animal de la Universidad de Zaragoza.

## Author Contributions

NA, EP, ER, FV, and CM designed the experiments and directed the study. NA, SU, EM, RT, DoM, AG, IO, RV, and CS performed the experiments. MM and JB provided material and facilities crucial for the execution of the experiments. NA, DeM, EP, FV, and CM wrote the manuscript. All authors contributed to the article and approved the submitted version.

## Conflict of Interest

NA, SU, DeM, EP, ER, and CM are co-inventors of the patent “inactivated tuberculosis vaccine” filled by the University of Zaragoza and Biofabri. The remaining authors declare that the research was conducted in the absence of any commercial or financial relationships that could be construed as a potential conflict of interest. The handling editor declared a past co-authorship with one of the authors CM.
