# Supplementary material for: Respiratory Immunization With a Whole Cell Inactivated Vaccine Induces Functional Mucosal Immunoglobulins Against Tuberculosis in Mice and Non-human Primates
Source: Front Microbiol. 2020 Jun 18;11:1339. doi: 10.3389/fmicb.2020.01339 (PMC7315045; doi:10.3389/fmicb.2020.01339)
Supplement: Supplementary file 1 [file Data_Sheet_1.DOCX]

SUPPLEMENTARY MATERIAL

**Supplementary Figure 1. MTBVAC appearance following heat treatment.** Representative electronic microscope images for live (left) and heat-killed (right) are shown in the figure.

**Supplementary Figure 2**. **Protective efficacy comparison using different methods of inactivation for MTBVAC.** Groups of C57BL/6 adult mice where vaccinated with BCG and inactivated MTBVAC by heat (HK) or formalin (FMLK) four weeks apart (10^7^ inactivated MTBVAC dose). After one month, mice were intranasally challenged with a low-dose H37Rv and lung bacterial load analyzed one month later. Data are shown as mean±SEM and are a pool of two independent experiments. (n=12 mice/group). *, p<0.05; by one-way ANOVA and Bonferroni post-test.

**Supplementary Figure 3**. **MTBVAC HK-induced protection in DBA/2 mice.** Groups of DBA/2 adult mice where vaccinated with BCG and 10^7^ MTBVAC HK four weeks apart. After one month, mice were intranasally challenged with a low-dose H37Rv and lung bacterial load analyzed one month later. Data are shown as mean±SEM from one experiment. (n=6 mice/group). **, p<0.01; by one-way ANOVA and Bonferroni post-test.

**Supplementary Figure 4. MTBVAC HK lot-to-lot comparison.** Groups of C57BL/6 adult mice where vaccinated with BCG and MTBVAC HK four weeks apart (three different lots, 10^7^ MTBVAC HK dose). After one month, mice were intranasally challenged with a low-dose H37Rv and lung bacterial load analyzed one month later. Data are shown as mean±SEM from one experiment. (n=6 mice/group). *, p<0.05; **, p<0.01; by one-way ANOVA and Bonferroni post-test.

**Supplementary Figure 5.** (**A**) Non-human primate IFNg response by ELISPOT over the 25-weeks vaccination phase. Group median values are plotted for non-vaccinated controls (non-v), standard BCG, and standard BCG plus MTBVAC HK boosted rhesus macaques. (**B-C**) Bacterial burden is enumerated post-mortem, 12 weeks after infectious challenge with *M.tuberculosis*, for hilar, lung-draining lymph nodes and spleen, respectively. (**D-G**) Changes in clinical parameters from infectious challenge to endpoint, 12 weeks post-infection, are plotted for body weight, serum C-reactive protein level, and mean corpuscular hemoglobin (MCH) and volume (MCV), respectively. Individual data points are consistently coloured according to the Supplemental Table 1; fat horizontal lines indicate group medians.

**Supplementary Figure 6.** Gating strategy to define CD4+ and CD8+ T lymphocytes in NHP BAL samples.

**Supplementary Table 1.** Adult healthy rhesus macaques were selected and stratified into non-vaccinated control and standard intradermal BCG vaccinated control groups, and a group that is boosted with mucosal MTBVAC HK after BCG. Relevant individual identifiers and characteristics are listed per treatment group.
