# Supplementary figures and images for: Respiratory Immunization With a Whole Cell Inactivated Vaccine Induces Functional Mucosal Immunoglobulins Against Tuberculosis in Mice and Non-human Primates
Source: Front Microbiol. 2020 Jun 18;11:1339. doi: 10.3389/fmicb.2020.01339 (PMC7315045; doi:10.3389/fmicb.2020.01339)

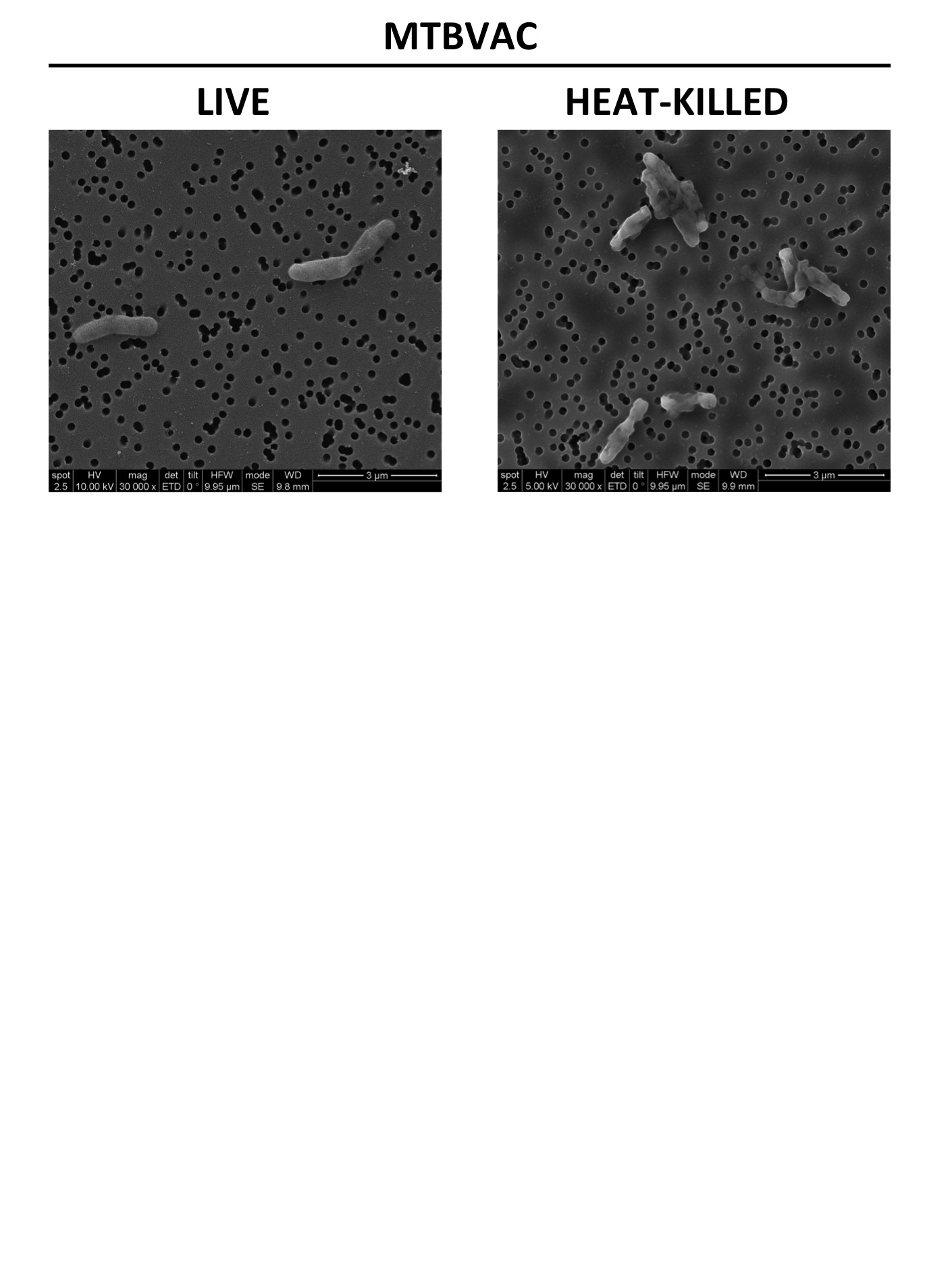

Supplement: Supplementary file 2 [file Image_1.JPEG]

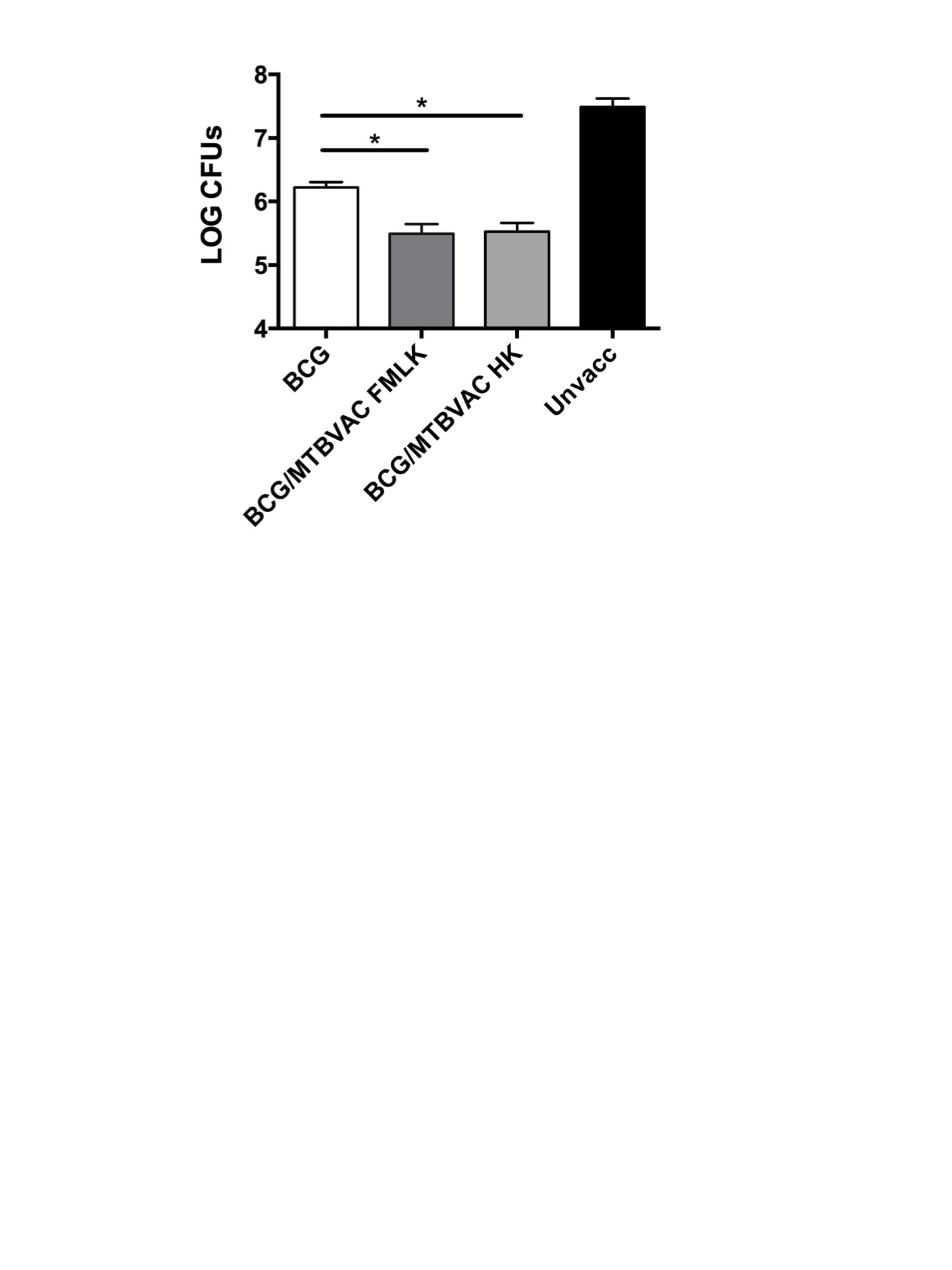

Supplement: Supplementary file 3 [file Image_2.JPEG]

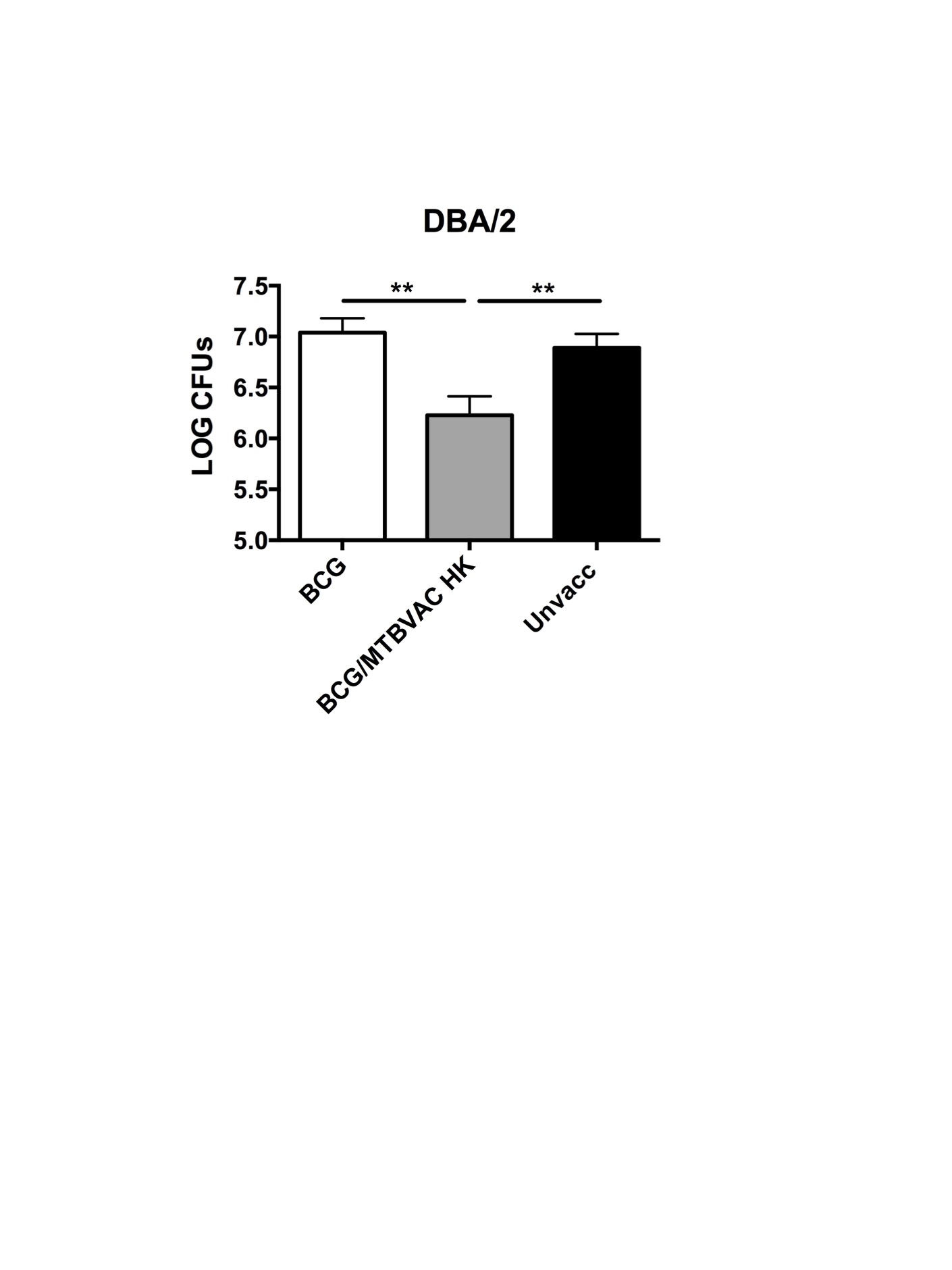

Supplement: Supplementary file 4 [file Image_3.JPEG]

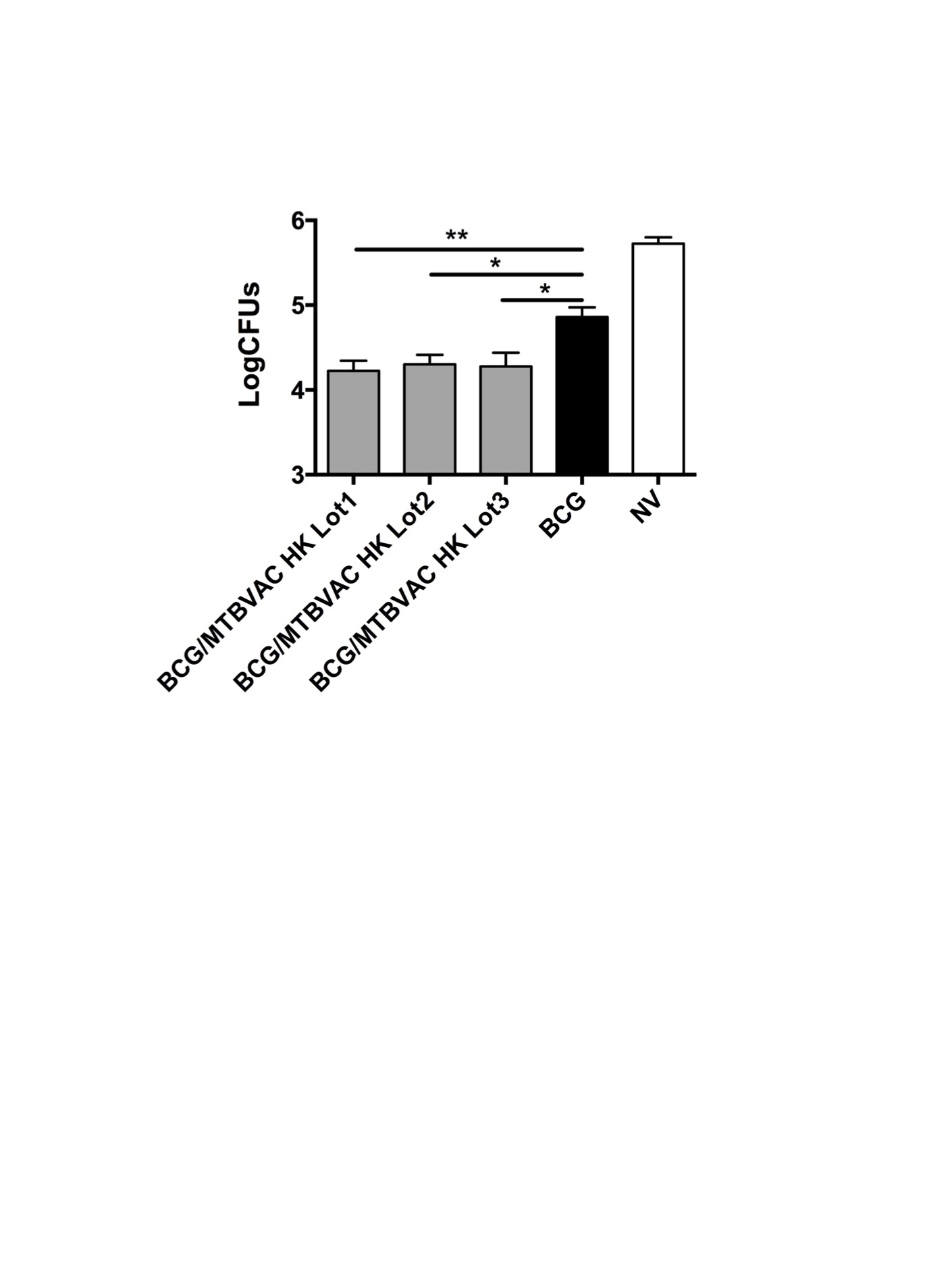

Supplement: Supplementary file 5 [file Image_4.JPEG]

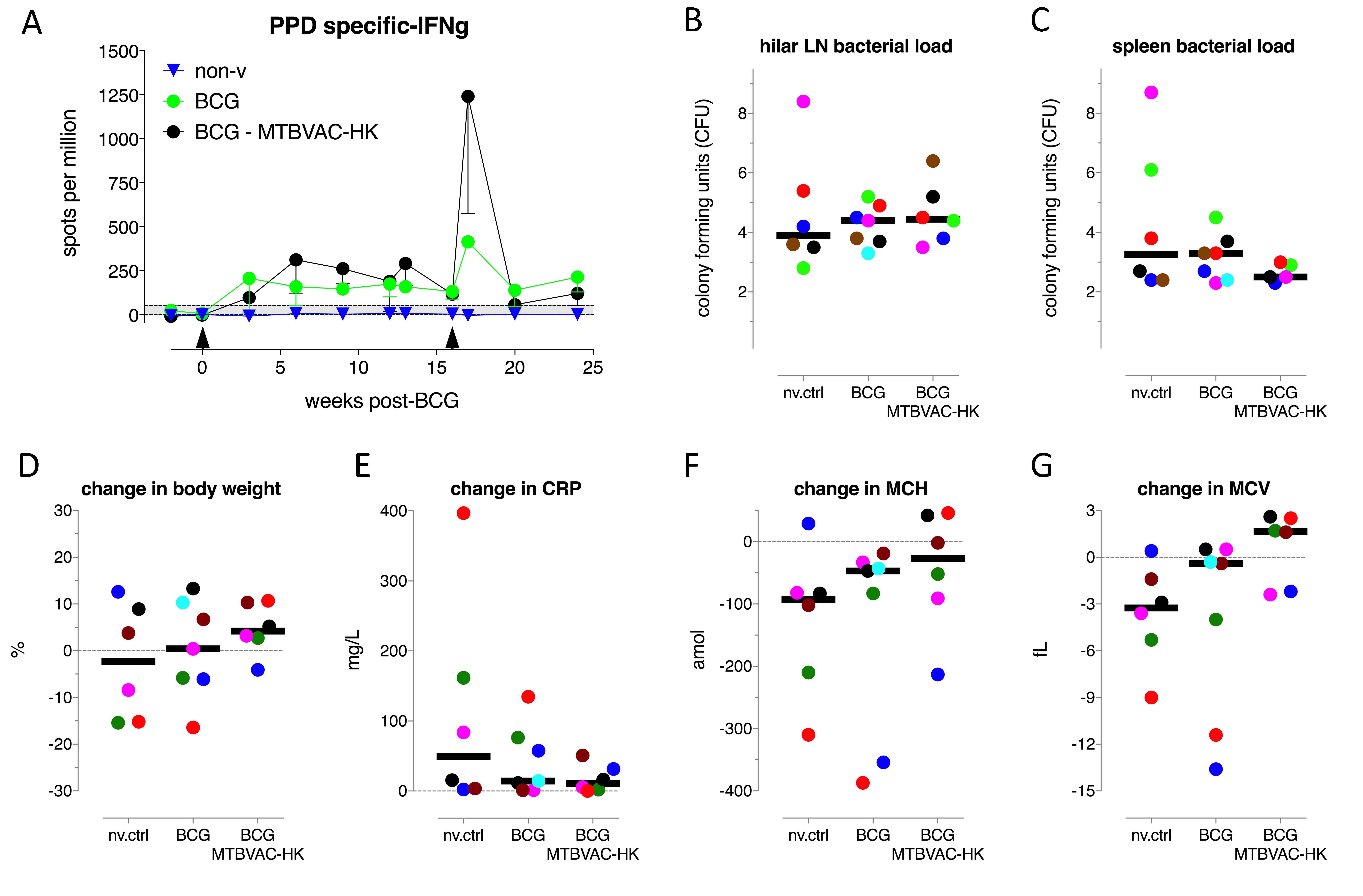

Supplement: Supplementary file 6 [file Image_5.JPEG]

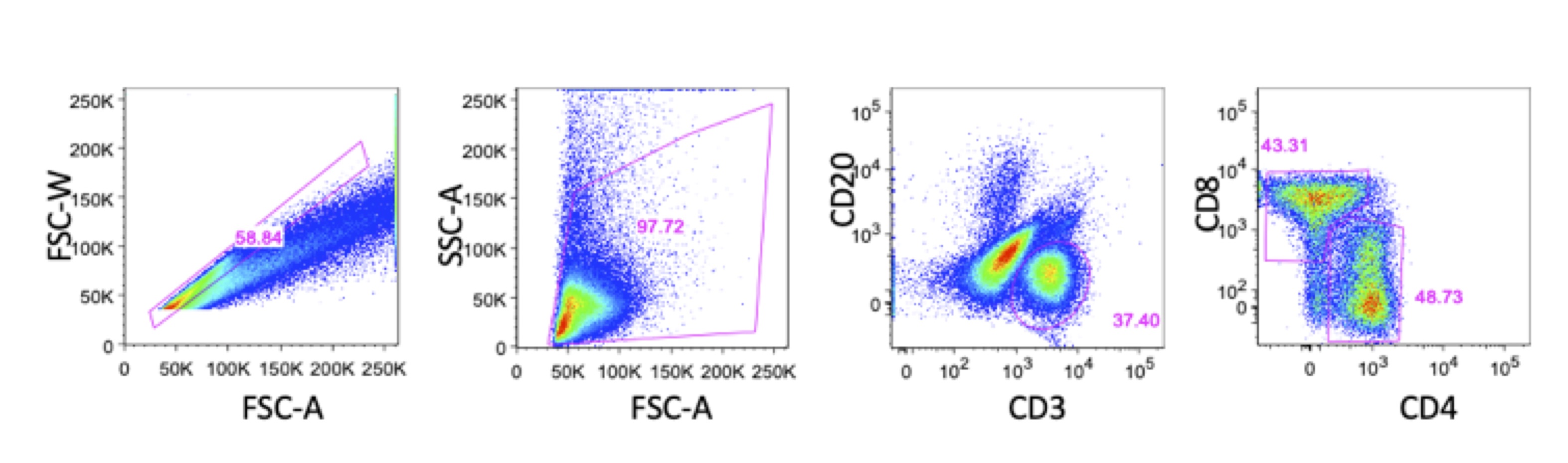

Supplement: Supplementary file 7 [file Image_6.JPEG]

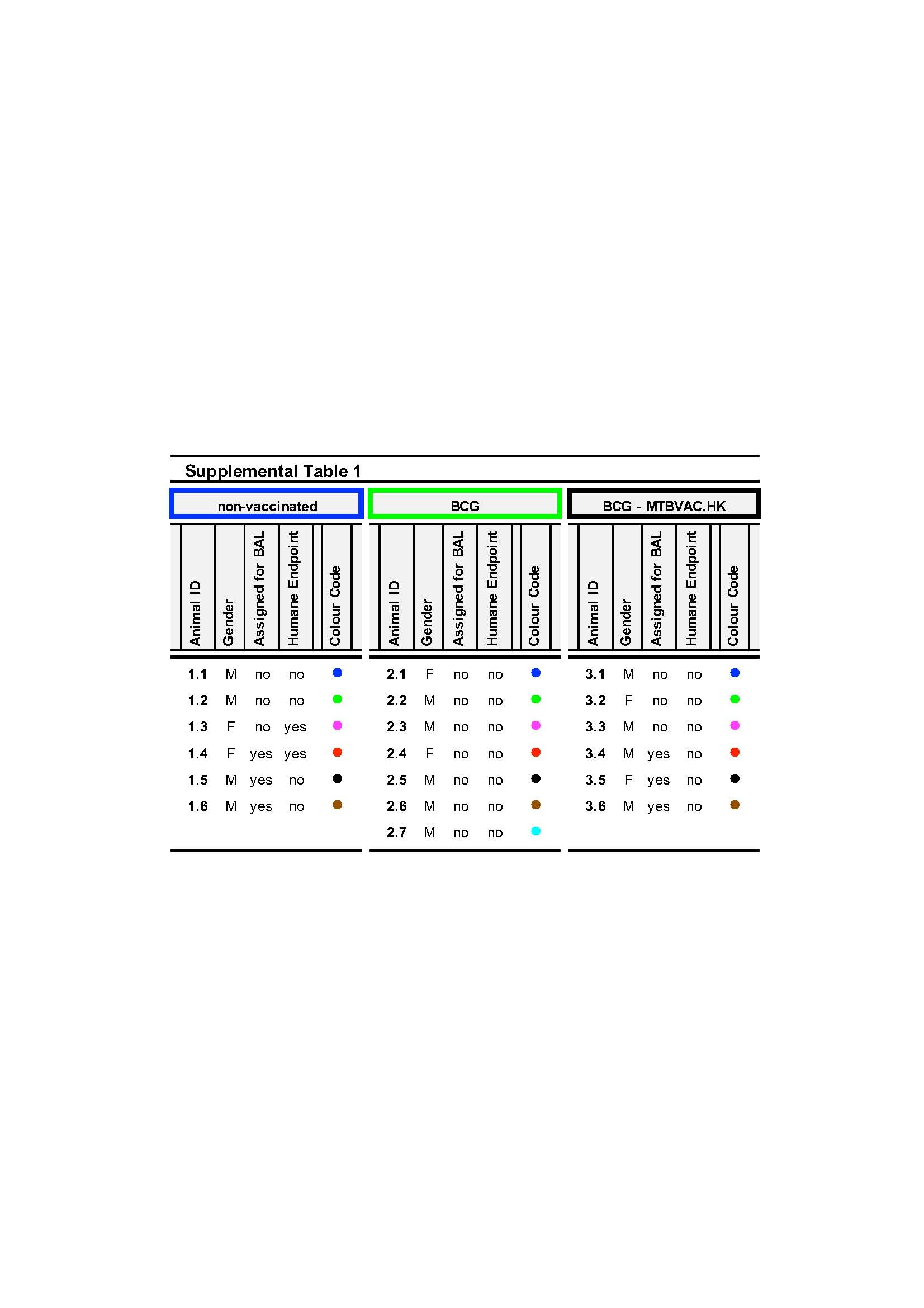

Supplement: Supplementary file 8 [file Image_7.JPEG]
